# Understanding the dynamics and interplay of public support and adherence to five key mitigation behaviors over the course of the evolving COVID-19 pandemic

**DOI:** 10.1016/j.ssmph.2025.101847

**Published:** 2025-07-24

**Authors:** Frank C. Gootjes, John B.F. de Wit, Denise D.T. de Ridder, F. Marijn Stok, Floor M. Kroese, Marijn de Bruin

**Affiliations:** a Programme Group Challenges to Democratic Representation, Faculty of Social and Behavioural Sciences, University of Amsterdam, PO Box 15578, 1001, NB Amsterdam, the Netherlands; b Department of Interdisciplinary Social Science, Utrecht University, PO Box 80140, 3508, TC Utrecht, the Netherlands; c Centre for Social Research in Health, UNSW Sydney, Goodsell Building, Sydney, NSW, 2052, Australia; d Department of Social, Health and Organizational Psychology, Utrecht University, PO Box 80140, 3508, TC Utrecht, the Netherlands; e National Coordination Infectious Disease Control, National Institute for Public Health and Environment, PO Box 1, 3720, BA Bilthoven, the Netherlands; f Centre for Prevention, Lifestyle, and Health Department Behaviour and Health, National Institute for Public Health and Environment, PO Box 1, 3720, BA Bilthoven, the Netherlands; g IQ Health, Radboud University Medical Centre, PO Box 9101, huispost 160, 6500, HB Nijmegen, the Netherlands

**Keywords:** COVID-19 mitigation measures, Policy support, Adherence, Cohort study, Cross-lagged panel analysis

## Abstract

Behavioral measures played a critical role in mitigating the COVID-19 pandemic, and their success hinged on continued public support and adherence. This study provides novel evidence on changes in support and adherence to behavioral mitigation measures and appraises the role of pandemic fatigue to provide robust guidance for effective governance of future public health and safety crises. Data were collected from a population cohort study in the Netherlands. This study used data from six assessments (December 2020–March 2022) at 12-week intervals, aligned with differences in pandemic severity and policy stringency. The analytic sample consisted of participants (N = 20,475) randomly allocated to answer questions on support and adherence to measures, focusing on physical distancing, avoiding crowds, mask-wearing on public transport, COVID-19 testing when symptomatic, and staying home when symptomatic. Changes in adherence and support and their interplay across time were assessed using random intercept cross-lagged panel models, controlling for age, gender and education. At the end of 2020, support for mitigation measures was found to be high, with little difference between measures. Subsequent changes in support for most measures broadly paralleled changes in pandemic severity and policy stringency. Adherence was less responsive to pandemic severity and policy stringency, and was mostly stable, albeit with differences between behavioral measures. Support and adherence to COVID-19 testing steadily increased after this was recommended as of early 2021. Changes in support and adherence did not reflect the notion of pandemic fatigue as a monotonic decline in support and adherence across behaviors. Findings highlight the need to better understand and address the factors influencing differing dynamics in support and adherence to specific protective behaviors.

## Highlights

• Public support and adherence did not steadily decline over time; but changes followed shifts in pandemic severity.
• Support tracked pandemic trends, but adherence stayed relatively stable and varied across behaviors.
• Support and adherence to testing rose steadily, likely due to self-test availability and policy recommendations.
• Men and younger people showed lower support and adherence; higher education was linked to better adherence to most measures.
• Support was not a consistent predictor of adherence and sometimes the reverse was true, especially during high-risk periods.

## 1 Introduction

The COVID-19 pandemic posed unprecedented challenges to global public health, and governments worldwide implemented a diversity of institutional and behavior change interventions to curtail the spread of the virus (Lee et al., 2021). A recent analysis of the time-varying effects of these mitigation measures in the United States estimated that the various measures implemented at different points in time contributed to more than half of reductions in the virus’ reproductive number in the early phase of the pandemic (Wu et al., 2024), with largest contributions made by international travel restrictions, stay-at-home requirements, and restrictions on gathering size. Mitigation measures were found to have remained critical as vaccination rates increased and plateaued (Wu et al., 2024), although their relative contribution decreased.

The success of mitigation measures hinged upon their acceptance by the general public (e.g., Margraf et al., 2020; Siegrist & Bearth, 2021), and the continued adherence to behavioral prevention measures has been identified as a key challenge (de Bruin et al., 2022). Already early in the COVID-19 pandemic, public health authorities and governments were concerned that the public would quickly experience fatigue with restrictions and only be able to maintain support and adherence to mitigation measures for a short period of time (see Harvey, 2020). The World Health Organization defined this potential pandemic fatigue as “demotivation to follow recommended protective behaviors, emerging gradually over time and affected by a number of emotions, experiences and perceptions” (WHO, 2020, p. 4).

Based on a review and synthesis of 30 studies on pandemic fatigue, Chen et al. (2024) distinguished two types of definitions of pandemic fatigue. Information and behavioral fatigue refers to the possible occurrence over time of a combination of emotions, experiences, and perceptions that may gradually lead to a decreased motivation to adhere to protective behaviors. Physical and mental fatigue reflect a decline in physical and/or mental abilities caused by alterations in central, psychological, and/or peripheral factors resulting from the COVID-19 disease. Furthermore, Chen et al. (2024) distinguished between assessments of subjective feelings of pandemic fatigue and assessments of changes in adherence to protective behaviors and noted that data were mostly collected through self-report surveys or recorded mobility data (Chen et al., 2024).

Behavioral evidence for a possible gradual decline in support and adherence to mitigation measures can be obtained from a number of studies that included assessments at two or more points in time. Some of these studies found decreases in support (Barry et al., 2021) or adherence (Brailovskaia et al., 2023; MacIntyre et al., 2021; Reinders Folmer et al., 2021; Sulemana et al., 2023). Other studies found a rebound in adherence after a decline (Brankston et al., 2022; Petherick et al., 2021). Also, several studies found stable levels of support that consistently differed between behavioral measures (De Wit et al., 2023; Foad et al., 2021). Furthermore, some studies found differences in changes in adherence for different behaviors (Du et al., 2022; Kim et al., 2023; Petherick et al., 2021; Smith et al., 2022), including increases in adherence (Kim et al., 2023; Levitt et al., 2022; Petherick et al., 2021; Smith et al., 2022). Extant studies on changes in support and adherence to COVID-19 behavioral measures provide mixed behavioral evidence on potential pandemic fatigue.

To count as indications of pandemic fatigue, decreases in support and adherence to pandemic mitigation measures should not be caused by other factors (Michie et al., 2020; Petherick et al., 2021). Alternative explanations for possible decreases in support and adherence have, however, been put forward that relate to people's life circumstances, including financial worries (Michie et al., 2020), the economic burden of mitigation measures, adjustments in risk perception, and the intensity of enforcement measures (Petherick et al., 2021). Mitigation measures differ in the psychological, social, or economic costs they may incur, and while adherence was found to decline for keeping safe physical distance (considered high cost), it increased for facemask wearing (considered low cost) (Petherick et al., 2021). Research has also highlighted the role of (evolving) perceived COVID-19 health threat in changes in support and adherence to mitigation measures, finding associations with perceived susceptibility, perceived severity, and experienced fear of COVID-19 (e.g., Chong et al., 2023; Foad et al., 2021; Kim et al., 2023; Reinders Folmer et al., 2021; Schumpe et al., 2022; Siegrist & Bearth, 2021). The perceived health risk of COVID-19 was found to be judged from the stringency of mitigation policy (Foad et al., 2021), which was adapted to evolving pandemic threat (De Bruin et al., 2022), notably to changes in hospitalizations because of COVID-19.

Concern about pandemic fatigue in the United Kingdom was invoked several times in 2020 to delay implementation or loosen restrictions (Reicher & Drury, 2021), which is estimated to have resulted in substantial numbers of additional deaths (cf. Harvey, 2020). Better understanding the dynamics of support and adherence to a diversity of pandemic measures remains critical to provide evidence-based guidance for policy makers to most effectively addressing future pandemics and other crises. The aim of this study is to undertake an in-depth analysis of the changes in support for and adherence to behavioral measures as the COVID-19 pandemic in the Netherlands evolved, and to appraise the possible role of pandemic fatigue in these changes. Our study is aligned with a behavioral definition of pandemic fatigue as a broad decline over time in support and adherence across mitigation behaviors. The study is guided by the expectation Petherick et al. (2021) derived from the WHO (2020) definition, that as behavioral mitigation measures continue, pandemic fatigue is reflected in a monotonic (linear or asymptotic) decrease in support and adherence across behavior that cannot be explained by other causes (cf. Petherick et al., 2021).

Our study extends the available research on support and adherence to COVID-19 mitigation measures in several ways. We report findings from a longitudinal cohort study and track changes over a prolonged period, from December 2020 to March 2022, controlling for possible differences in demographic characteristics (i.e., age, gender, and education). We simultaneously assess support and adherence to five critical and distinct mitigation behaviors: keeping appropriate physical distance, avoiding crowded places if keeping distance is not possible, wearing a face mask on public transport, testing for COVID-19 when experiencing symptoms, and staying at home when experiencing symptoms. We also jointly assess changes in support and adherence as well as their interplay, enabling an examination of how support and adherence to protective behaviors measures co-evolved and affected each other over time.

## 2 Methods

### 2.1 Design and participants

This study draws on data from a population cohort study in the Netherlands conducted by the National Institute for Public Health and the Environment (for a detailed description, see Van den Boom et al., 2023). The study was undertaken from April 2020 until June 2022 and collected data at 21 time points through online self-report surveys. Initial participants were recruited from research panels of all regional public health services in The Netherlands. Periodically, additional participants were recruited via social media campaigns. All participants who had completed a survey at any time point were invited to participate at every subsequent time point. For the present study, we used data from six assessments between December 2020 and March 2022, with 12 weeks between each time point. We selected the time points to focus on the period beyond the well-studied initial pandemic phase and limited the number of time points to reduce the complexity of the analyses and their interpretation. The included time points coincided with dynamics in pandemic severity, as reflected in the stringency of mitigation measures and COVID-19-related hospital admissions. Across the six selected time points, 95,275 unique individuals participated (N _time point 1_ = 53,571, N _time point 2_ = 50,120, N _time point 3_ = 52,374, N _time point 4_ = 44,366, N _time point 5_ = 46,441, N _time point 6_ = 37,503). To prevent overburdening participants, about one-third was randomly allocated by the survey tool to the questions on support and adherence to COVID-19 mitigation measures, resulting in a subset of 30,656 participants. To assess individual-level changes, the analytic sample was restricted to participants who answered the questions on support and adherence on at least two time points, resulting in the exclusion of about one third of participants. Of the participants for whom data could be analyzed (N = 20,475) 15 % participated in two, 14 % in three, 12 % in four, 11 % in five, and 14 % on all six time points.

### 2.2 Assessment

#### 2.2.1 Support for COVID-19 mitigation measures

Support for mitigation measures (keeping appropriate physical distance, avoiding crowded places, wearing a face mask on public transport, testing for COVID-19 when experiencing symptoms, and staying at home when experiencing symptoms) was assessed with a single item each. Responses were indicated on a 5-point scale (1 = not at all, 5 = completely). A ‘no opinion’ option was also provided and treated as a missing value.

#### 2.2.2 Adherence to COVID-19 mitigation measures

Adherence regarding keeping appropriate distance was assessed with five questions on how often people in the last week came closer than 1.5 m distance from others across five situations (last time going to work, last time going to shop, last time taking care of someone, last time going to a party, and last time receiving visitors at home or visiting family or friends). People who reported not having been in a specific situation were not presented with the question about keeping distance in that situation. An average score was calculated per person based on the situations they had been in. The average score was subsequently reverse coded so that a higher score reflected more adherence to this measure.

Adherence regarding avoiding places that are crowded was assessed with two items: “In the past 7 days (1 week), how often have you been in a place where it was too crowded to keep 1.5 m distance?”, and “How many of those [X] times did you turn around or go somewhere else because it was too crowded?”. As nearly all values (99.5 %) were below 11, responses were truncated at 11. An indicator of non-avoidance of crowded places was computed by subtracting the response to the question on turning around from the response to the question on have been in a crowed place. A score of zero was imputed if the participant had indicated no occasions of having been in a crowded place. Scores were subsequently reverse coded, with higher values reflecting more adherence (i.e., fewer times in crowded situations).

Adherence regarding wearing a facemask on public transport was assessed with a single item, asking if participants wore a face mask during the entire last ride they made using public transport. Response options included “yes”, “yes, but briefly took it off to eat or drink”, and “no”. Both yes responses were considered indicative of adherence. If people had not taken public transport in the preceding six weeks, they were not asked this question, and their responses were set to missing.

To determine if people adhered to testing for COVID-19 when experiencing symptoms, we asked if they had experienced relevant symptoms in the past six weeks and whether they thought these could be related to another health issue. Next, participants were asked if they had taken a COVID-19 test in the past 6 weeks because of these symptoms. Adherence was indicated as having done a test while experiencing symptoms that were not considered due to other health conditions.

Adherence to staying at home when experiencing symptoms was assessed by first establishing whether participants had experienced symptoms likely related to COVID-19 based on the above items. Participants were then asked to indicate if they had stayed at home because of these symptoms. People were considered to have adhered to the mitigation measure if they had relevant symptoms and had not been out of the house, had been out of the house only after a negative test result, or had been out of the house only to get tested.

### 2.3 Data analysis

Changes in adherence and support and their interplay across time points were assessed using cross-lagged panel analysis with a random intercept. The analyses examined the direction and strength of associations between adherence and support at two points in time, and the inclusion of a random intercept accounted for stability in adherence and support across assessments (see Hamaker et al., 2015; Lucas, 2023). The analyses separated between-person variance in support and adherence from within-level variance to prevent spurious cross-lagged effects and underestimation of true effects. Missing data were addressed by full-information-maximum-likelihood, producing accurate results under assumptions of missing-at-random (Lee & Shi, 2021). Scores for support, adherence, and age were normalized (minimum = 0, maximum = 1).

An RI-CLPM was run for every mitigation measure separately, including support and adherence at each time point as main variables. Age, gender and education were included as potential demographic control variables. The association between support and adherence and each of the demographic variables was constrained to be equal across time points, assuming associations are equal over time. The effect of each of the demographic variables was constrained to be equal across time points, assuming its effect on adherence and support is equal over time. Not all measures were available at each time point. Time point 6 did not include the support and adherence questions for avoiding crowded places, and time point 1 and 2 did not include the question about testing when experiencing symptoms. These time points were not included in the respective analyses.

**Fig. 1:**
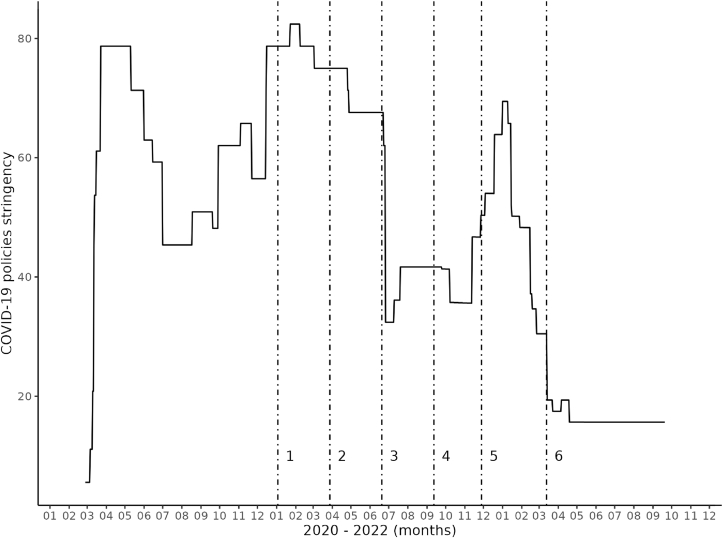
Policy Stringency Index scores during the COVID-19 pandemic in The Netherlands *Note:* Vertical dotted lines indicate data collection time points. Time point 1: December 30, 2020–January 3, 2021; time point 2: 24–March 28, 2021; time point 3: 16–June 20, 2021; time point 4: 8– 12 September 2021; time point 5: 24–November 28, 2021; time point 6: 9–March 13, 2022. Source: Roser (2021).

**Fig. 2:**
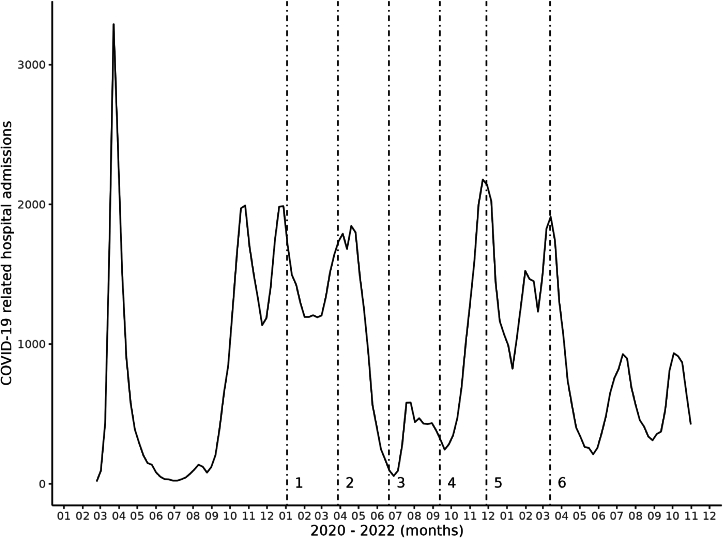
Number of COVID-19 related hospital admissions during in The Netherlands *Note:* Vertical dotted lines indicate data collection time points. Time point 1: December 30, 2020–January 3, 2021; time point 2: 24–March 28, 2021; time point 3: 16–June 20, 2021; time point 4: 8–September 12, 2021; time point 5: 24–November 28, 2021; time point 6: 9–March 13, 2022. Source: RIVM (2024).

The standardized estimated model parameters are interpreted in light of two sources of national-level information about the evolving pandemic in the Netherlands: the COVID-19 stringency index (Hale et al., 2021), and COVID-19-related hospital admissions. The COVID-19 stringency index (range 0–100) reflects how strict the COVID-19 policies in a country are. Data for The Netherlands was taken from the website ourworldindata.org (Roser, 2021); see Fig. 1. The data on the number of COVID-19-related hospital admissions was taken from the website of the National Institute for Public Health and Environment (RIVM, 2024); see Fig. 2.

## 3 Results

### 3.1 Sample characteristics

Of the participants included in the analyses, 36 % identified as male, 64 % as female, and 0.1 % as other (set to missing in the analyses due to the small proportion). In terms of age, 5 % of the sample was younger than 30 years, 29 % of the sample was between 30 and 50 years, 33 % was between 50 and 65 years, and 32 % was 65 years or older. According to the categories of Statistics Netherlands (Pleijers & de Vries, 2021), 11 % of participants had completed lower education, 26 % had completed middle-level education, and 63 % had completed higher education.

Compared to participants who answered questions on support and adherence to mitigation measures on only one time point, more participants included in the analysis were men (36 % vs 30 %), of older age (5 % vs 12 % younger than 30, 29 % vs 43 % older than 30 and younger than 50, 29 % vs 33 % older than 50 and younger than 65, 32 % vs 16 % people older than 65 years) and higher educated (63 % vs 57 %). Compared to the population of the Netherlands in 2020, based on data of Statistics Netherlands (see https://opendata.cbs.nl/statline/#/CBS/nl/), the analytic sample underrepresented men (36 % vs 49 %), persons younger than 30 (5 % vs 19 %), and those with a lower level of education (11 % vs 26 %). People over 65 years were overrepresented (32 % vs 24 %), as were those with a higher level of education (63 % vs 34 %).

### 3.2 Support and adherence to mitigation measures

**Fig. 3:**
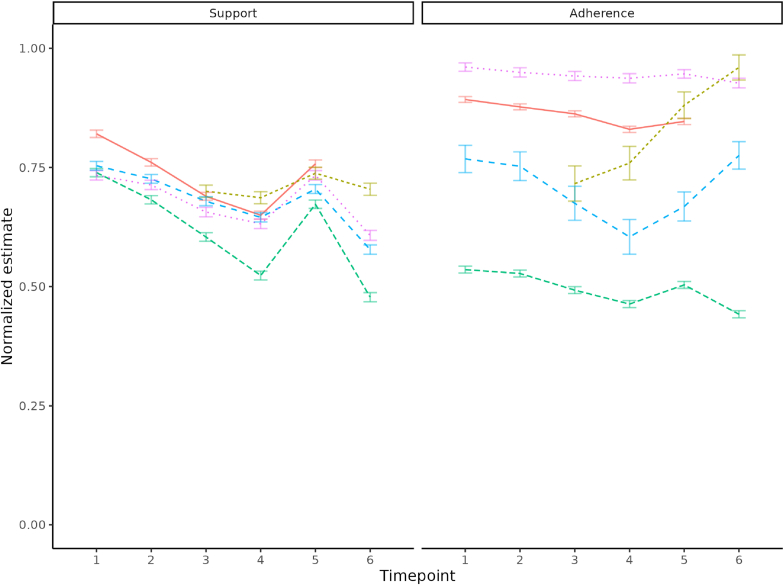
Estimated sample mean scores for support and adherence to COVID-19 mitigation measures *Legend:* red lines: avoiding crowded places; green lines: keeping appropriate physical distance; pink lines: wearing a facemask on public transport; beige lines: getting tested when experiencing COVID-19-related symptom; blue lines: staying at home when experiencing COVID-19-related symptoms. *Note:* Scores on support and adherence were normalized such that the minimum value was 0 and the maximum value 1.

As shown in Fig. 3 (left-hand panel), at time point 1, support for all assessed mitigation measures was similar and high (also see Supplementary Tables S1–S5). The RI-CLPM analyses showed that support declined between time points 1 and 4 for all measures, except for testing when experiencing symptoms. Support for all measures increased between time points 4 and 5, after which it declined for measures that were assessed at time point 6. The analyses were controlled for possible associations between demographic characteristics and support, modelled as time-invariant covariates. Men, compared to women, reported significantly lower support for all measures (parameters ranging from −0.044 to −0.115). Older age was significantly related to more support for all measures (parameters ranging from 0.084 to 0.258). Higher education, compared to middle-level education, was significantly associated with more support for all behavioral measures (parameters ranging from 0.025 to 0.079). Lower education, compared to middle-level education, was significantly associated with less support for keeping appropriate physical distance from others and avoiding crowded places (parameters ranging from −0.016 to −0.024) but not with support for other measures.

As shown in Fig. 3 (right-hand panel), at time point 1, there were differences in adherence to measures assessed (also see Supplementary Tables S1–S5). Adherence was highest for wearing a facemask on public transport and avoiding crowded places, moderate for staying at home when experiencing symptoms and lowest for keeping appropriate physical distance. The rank order in adherence to these four measures was consistent over time. The RI-CLPM analyses showed that adherence to avoiding crowded places and wearing a facemask on public transport remained high but declined somewhat from time point 1 to time point 6. Adherence to keeping appropriate physical distance declined further from an already low at time point 1, despite a temporary increase at time point 5. Adherence to staying at home when experiencing symptoms fluctuated, with a decline between time points 1 and 5, followed by an increase. Changes in adherence to testing when experiencing symptoms were dissimilar and continued to increase. RI-CLPM analyses of adherence also accounted for demographic characteristics as time-invariant covariates.

Men reported significantly less adherence to each of the measures than women (parameters ranging from −0.020 to −0.133). Older age was significantly associated with higher adherence to keeping appropriate physical distance, avoiding crowded places, and wearing a facemask on public transport (parameters ranging from 0.066 to 0.215), while it was significantly associated with lower adherence to getting tested and staying at home when experiencing COVID-19-related symptoms (parameters ranging from −0.043 to −0.096). Higher education, compared to middle-level education, was significantly associated with more adherence to all measures (parameters ranging from 0.032 to 0.050), except wearing a facemask on public transport, for which no association was found. Lower education, compared to middle-level education, was significantly associated with more adherence to keeping appropriate physical distance (parameters ranging from 0.027 to 0.030) but not with adherence to other measures.

### 3.3 Interplay of support and adherence

#### 3.3.1 Keeping appropriate physical distance

Means, standard deviations, and correlations among assessments of support and adherence to keeping appropriate physical distance are shown in Supplementary Table S6. The results of the RI-CLPM analysis are shown in Supplementary Fig. S1. The analysis found substantial stability in differences between participants’ support and adherence to keeping appropriate physical distance over time, as reflected in 50 % of variance in support and 48 % of variance in adherence attributed to between-person variance. Between-person variance in support and adherence were significantly correlated (*r* = 0.41, *p* < .001). At the within-person level, results show that cross-lagged paths were generally significant; support generally was associated with adherence at the subsequent time point, and vice versa, adherence was generally associated with support at a subsequent time point. The results further suggest that support is a stronger precursors of adherence than vice versa at time points when pandemic threat was relatively low or decreasing. Notably, support was more strongly associated with subsequent adherence from time points 3 to 4, when policy stringency and hospital admissions were lower, and from time points 5 to 6 when policy stringency decreased, although numbers of hospitalizations were high. Adherence was more strongly associated with subsequent support than vice versa when pandemic severity was high or increasing (from time points 2 to 3, and from time points 4 to 5).

#### 3.3.2 Avoiding crowded places

Means, standard deviations, and correlations among assessments of support and adherence to avoiding crowded places are shown in Supplementary Table S7. The results of the RI-CLPM analysis are shown in Supplementary Fig. S2. This showed that 49 % of the variance in support and 40 % of the variance in adherence could be attributed to stable between-person differences. The between-person variances in adherence and support were significantly correlated (*r* = 0.44, *p* < .001). Cross-lagged paths between adherence and support were generally significant. At the within-person level, support was more strongly associated with subsequent adherence than vice versa when policy stringency and pandemic severity were lower (from time point 3 to 4), while adherence was more strongly associated with subsequent support than vice versa when the pandemic was changing (decreasing between time point 2 to 3 and increasing between time point 4 to 5).

#### 3.3.3 Wearing a facemask on public transport

Means, standard deviations, and correlations among assessments of support and adherence to wearing a facemask on public transport are shown in Supplementary Table S8. The results of the RI-CLPM analysis are depicted in Supplementary Fig. S3 and indicated that the models did not have an optimal fit. This is likely because this measure was closely monitored, and most people using public transport consequently adhered to it, irrespective of their support. Between-person variance in support (53 % of total variance) and adherence (43 % of total variance) were significantly correlated (*r* = 0.56, *p* < .001). At the within-person level, cross-lagged paths between support and adherence were generally not significant. An exception is that support at time point 4 was negatively associated with subsequent adherence at time point 5. Adherence at time point 5 was negatively associated with subsequent support at time point 6, while support at time point 5 was positively associated with adherence at time point 6.

#### 3.3.4 Getting tested when experiencing symptoms

Means, standard deviations, and correlations among assessments of support and adherence to getting tested when experiencing COVID-19-related symptoms are shown in Supplementary Table S9. The RI-CLPM analysis (see Supplementary Fig. S4) found that the between-person variance in support (61 % of total variance) and adherence (19 % of total variance) were significantly correlated (*r* = 0.57, *p* < .001). The proportion of between-person variance in adherence was lower compared to other measures, suggesting less stable between-person differences in adherence over time than for other measures. At the within-person level, cross-lagged paths between support and adherence were generally not significant, except for adherence at time point 5, which was significantly associated with support at time point 6. At each time point, all correlations between support and adherence were significant and positive (see Supplementary Table S9).

#### 3.3.5 Staying at home when experiencing symptoms

Means, standard deviations, and correlations among assessments of support and adherence to staying at home when experiencing COVID-19-related symptoms are shown in Supplementary Table S10. The results of the RI-CLPM analysis are depicted in Supplementary Fig. S5. The between-person variance in support (50 % of total variance) and adherence (19 % of total variance) were significantly correlated (*r* = 0.51, *p* < .001). The limited between-person variance again suggests low and stable differences in adherence. At the within-person level, cross-lagged paths between support and adherence were generally not significant. The exception was that adherence at time point 1 was significantly associated with support at time point 2. The correlation between support and adherence within time points was, nevertheless, significant and positive at all time points (see Supplementary Table S10).

## 4 Discussion

Public support and adherence are crucial for the effectiveness of COVID-19 behavioral mitigation measures (e.g., Margraf et al., 2020; Siegrist & Bearth, 2021). However, how this support evolved over time—particularly after the initial crisis and into the second year of the pandemic—remains understudied. It is also unclear whether concerns about pandemic fatigue are born out (Lilleholt et al., 2023; Petherick et al., 2021), or whether any changes in support or adherence could reflect other processes (De Wit et al., 2023; Waterschoot et al., 2023). Understanding the dynamics in support and adherence is essential for designing crisis governance strategies that balance public health with the resumption of societal functions (Lazarus et al., 2023).

We found high support for mitigation measures in the Netherlands in December 2020 – roughly one year after the initial outbreak in Wuhan (Amodio et al., 2020) and 9–10 months after the first national case (Rijksoverheid, 2020). This included support for physical distancing, avoiding crowded places, mask use on public transport, and staying home when symptomatic. Differences between these measures were minimal at that point. Throughout 2021 and early 2022, support for mitigation measures shifted largely in line with perceived pandemic threat, as indicated by policy stringency and number of hospitalizations. Support declined as the threat subsided in 2021, rose again with the emergence of the Omicron variant in late 2021, and fell once more as the pandemic situation improved in early 2022. By the end of 2021, support varied between measures. Testing when symptomatic had the highest support, while physical distancing had the lowest. Notably, support for testing increased steadily, likely due to the introduction and uptake of rapid antigen self-tests, which became recommended in the Netherlands from December 2, 2021 (Rijksoverheid, n.d.). These findings build on earlier evidence from the U.S. (Barry et al., 2021), where support initially declined and then stabilized between April and November 2020. This study also extends our prior research in the Netherlands covering April 2020 to May 2021 (De Wit et al., 2023), which found high, stable support for measures like hygiene, mask use, and isolation, declining support for travel restrictions and limits on home visitors, and increasing support for limiting visitors at home when perceived risk increased (De Wit et al., 2023).

Adherence to mitigation measures followed a different pattern. Unlike support, adherence generally did not mirror changes in pandemic threat. Instead, adherence varied more consistently by behavior. It was highest and most stable for mask use on public transport and avoiding crowds. Physical distancing had the lowest and gradually declining adherence. Staying home when symptomatic showed intermediate adherence, with some responsiveness to rising threat levels. Testing adherence increased steadily, mirroring the rise in support and likely driven by the availability of self-testing options. These findings are consistent with other cohort studies showing diverse trajectories across protective behaviors—ranging from overall declines (Sulemana et al., 2023), to non-linear changes (Brankston et al., 2022), to sustained or increasing adherence for some measures (Kim et al., 2023; Levitt et al., 2022; Smith et al., 2022).

Analyses were controlled for age, gender and education and findings showed that support and adherence to behavioral mitigation measures differed between demographic subgroups. Men were less likely to support and adhere to measures than women. Older people were more likely to support all measures and adhere to some measures (i.e., keeping appropriate physical distance, avoiding crowded places, and wearing a facemask on public transport), while they were less likely to adhere to others (i.e., getting tested and staying at home when experiencing COVID-19-related symptoms). We found few differences in support of measures related to education. We did, however, find that higher educated people were more likely to adhere to measures, except wearing a facemask on public transport. Lower education was associated with more adherence to keeping appropriate physical distance. Prior research has also noted differences in support and adherence to mitigation measures related to age (e.g., Barry et al., 2021; De Wit et al., 2023; Levitt et al., 2022; Thomas et al., 2023), gender (Sulemana et al., 2023; Thomas et al., 2023) and education (Kim et al., 2023). These differences in support and adherence between demographic groups remain to be understood, which is beyond the scope of this study due to a lack of data on possible explanatory processes. Michie et al. (2020) have suggested that differences between demographic subgroups likely reflect people's diverse life circumstances, and the important role of structural factors has also been noted (e.g., Reicher & Drury, 2021).

We found differences in time-lagged associations between support and adherence to the mitigation measures. Associations between support and adherence across subsequent time points were generally significant for keeping appropriate physical distance and avoiding crowded place, while these were generally not significant for support and adherence to getting tested and staying at home when experiencing symptoms. Only some significant time-lagged associations were found between support and adherence to wearing a facemask on public transport. Also, when adherence and support were significantly associated over time, adherence mostly was as strongly or more strongly associated with subsequent support than vice versa. Measures of public support hence do not seem an optimal indicator of the (potential) adherence to implemented mitigation measures. The limited associations between support and adherence over time may reflect different underlying processes. As Foad et al. (2021) have found in the United Kingdom, people's support for mitigation policies was significantly associated with their perceived health risk of COVID-19, which they judged from the stringency of mitigation policy. Adherence to mitigation behaviors, in contrast, is thought to be associated with diverse factors affecting not only people's motivation but also their capacity and opportunity to engage in the protective behavior (e.g., Michie & West, 2021; West et al., 2020).

Our findings highlight the importance of policy makers and intervention designers focusing on promoting adherence to mitigation measures in ways that go beyond ensuring support for such measures. This requires understanding the specific factors that may affect adherence to distinct protective behaviors for people in different life circumstances (Michie & West, 2021). Such advanced behavioral understanding is critical to develop targeted prevention approaches that are required to maximize success (e.g., Sulemana et al., 2023). A systematic review of interventions to increase a range of personal protective behaviors found low-quality evidence for positive effects of interventions targeting hand hygiene, unclear results for interventions targeting face mask use, and a lack of evidence for most behaviors of interest (Perski et al., 2023). This limited evidence underscores the importance of further research and related investment to test and develop behavior change approaches to effectively promote protection against COVID-19 and other pandemic outbreaks. The potential success of behavioral intervention can be harnessed by drawing on systematic approaches, such as the behavior change wheel (see Michie & West, 2021; West et al., 2020). This enables intervention designers to link understanding of the factors affecting people's capability, opportunity and motivation to enact a behavior to the types of intervention that are most likely to be effective and choose appropriate implementation (Michie & West, 2021; West et al., 2020).

The reported study goes beyond prior research by prospectively assessing support and adherence for mitigation measures as well as their interplay for diverse behavioral measures in a diverse sample of the population across multiple time points differing in pandemic threat and policy stringency. However, the study also has several limitations. The study sample was large and diverse, but not fully representative of the population in the Netherlands, as men, younger people and people who completed lower and middle-level education were underrepresented. Also, data were collected through self-report and could have been affected by memory and social desirability biases, albeit that the surveys were anonymous. Support and adherence to mitigation behaviors were asked with single items, which precluded a more comprehensive and nuanced assessment and may have limited the reliability of measures. Also note that participants who participated once were excluded, and the reported support and adherence of these possibly more motivate participants may be an overestimation compared to the population. Furthermore, while the prospective design enabled the assessment of changes and associations across time points, findings do not provide evidence of causal relations. Also, some change dynamics might have been missed in our analysis as time points were twelve weeks apart, during which period the stringency of measures and number of hospitalizations might have changed. Relatedly, the period we analyzed only started at the end of 2020, during the second major wave of infections in the Netherlands, and dynamics in the earlier phases of the pandemic could have been different.

Do our findings provide evidence for the role of pandemic fatigue in changes in support and adherence to five distinct COVID-19 prevention measures? Drawing on the WHO (2020) definition of pandemic fatigue as demotivation to follow recommended protective behaviors that emerges gradually over time, pandemic fatigue is expected to manifest as a monotonic decline behavioral engagement, as longs as mitigation measures are in place and that cannot be explained by other causes (cf. Petherick et al., 2021; WHO, 2020). We did not find monotonic declines in support or adherence to mitigation measures. We did find that support for behavioral measures declined between December 2020 and September 2021, but this coincided with a decrease in stringency of COVID-19 protection policies and numbers of hospitalizations. This suggests that reduced pandemic threat, rather than pandemic fatigue, is a likely alternative explanation for the observed decrease in support to mitigation behaviors. The responsiveness of support for mitigation measures is also reflected in a subsequent increase, coinciding with more strict policies and higher numbers of hospitalizations. Furthermore, rather than a gradual decline, we found that adherence was largely stable. Also, adherence consistently differed between mitigation behaviors. which has been attributed to potential differences in the costs of behaviors, with sustained adherence more likely for low-cost than high-cost behaviors (Petherick et al., 2021). It has also been found that support and adherence to behavioral measures are contingent on the proportionality of policy stringency relative to pandemic threat (Waterschoot et al., 2023). This suggests that people may engage in an evolving trade-off between perceived costs and benefits of protective behaviors that likely differs according to people's life circumstances (cf. Michie et al., 2020; Reicher & Drury, 2021).

The lack of behavioral evidence for pandemic fatigue does not preclude that people may subjectively experience a sense of pandemic fatigue. A study reporting the development of the pandemic fatigue scale found that subjectively experienced pandemic fatigue increased over time in cross-sectional and panel studies in Germany and Denmark undertaken between October 2020 and September 2021 (Lilleholt et al., 2023). However, while this study found significant associations between the subjective experience of pandemic fatigue and adherence to behavioral mitigation measures, these were generally small or moderate at best, and smaller than associations between adherence and other factors, especially perceived risk of COVID-19 (Lilleholt et al., 2023). These findings suggest that the two approaches to pandemic fatigue distinguished by Chen et al. (2024) may reflect at least partially distinct phenomena, with partial overlap of subjective experiences and behaviors.

## CRediT authorship contribution statement

**Frank C. Gootjes:** Writing – review & editing, Writing – original draft, Visualization, Methodology, Formal analysis, Data curation, Conceptualization. **John B.F. de Wit:** Writing – review & editing, Writing – original draft, Supervision, Methodology, Funding acquisition, Conceptualization. **Denise D.T. de Ridder:** Writing – review & editing, Writing – original draft, Supervision, Methodology, Funding acquisition, Conceptualization. **F. Marijn Stok:** Writing – review & editing, Writing – original draft, Supervision, Methodology, Conceptualization. **Floor M. Kroese:** Writing – review & editing, Writing – original draft, Supervision, Methodology, Conceptualization. **Marijn de Bruin:** Writing – review & editing, Writing – original draft, Validation, Project administration, Funding acquisition.

## Ethical statement

The study protocol was evaluated by the Centre for Clinical Expertise of the National Institute for Public Health and the Environment (RIVM) of the Netherlands (study number G&M-516). The study protocol was considered exempt from medical ethics review as governed by the Law on Research Involving Human Subjects (WMO) in The Netherlands and approved for implementation. The study was conducted in accordance with the Declaration of Helsinki of the World Medical Association and adhered to the General Data Protection Regulation and relevant codes of conduct of participating institutions. All participants provided informed consent for participation prior to completion of their first survey. After completing the first survey they provided informed consent for participation in the cohort study.

## Declaration of competing interest

The authors have no (potential or perceived) conflicting interests to declare.

## Data Availability

Data will be made available on request.
