# Supplementary material for: Understanding the dynamics and interplay of public support and adherence to five key mitigation behaviors over the course of the evolving COVID-19 pandemic
Source: SSM Popul Health. 2025 Jul 24;31:101847. doi: 10.1016/j.ssmph.2025.101847 (PMC12314321; doi:10.1016/j.ssmph.2025.101847)
Supplement: Multimedia component 1 [file mmc1.docx]

**Understanding the dynamics and interplay of public support and adherence to five key mitigation behaviors over the course of the evolving COVID-19 pandemic**

# Supplementary materials

## Figure S1

*RI-CLPM analysis of support and adherence regarding keeping appropriate physical distance*


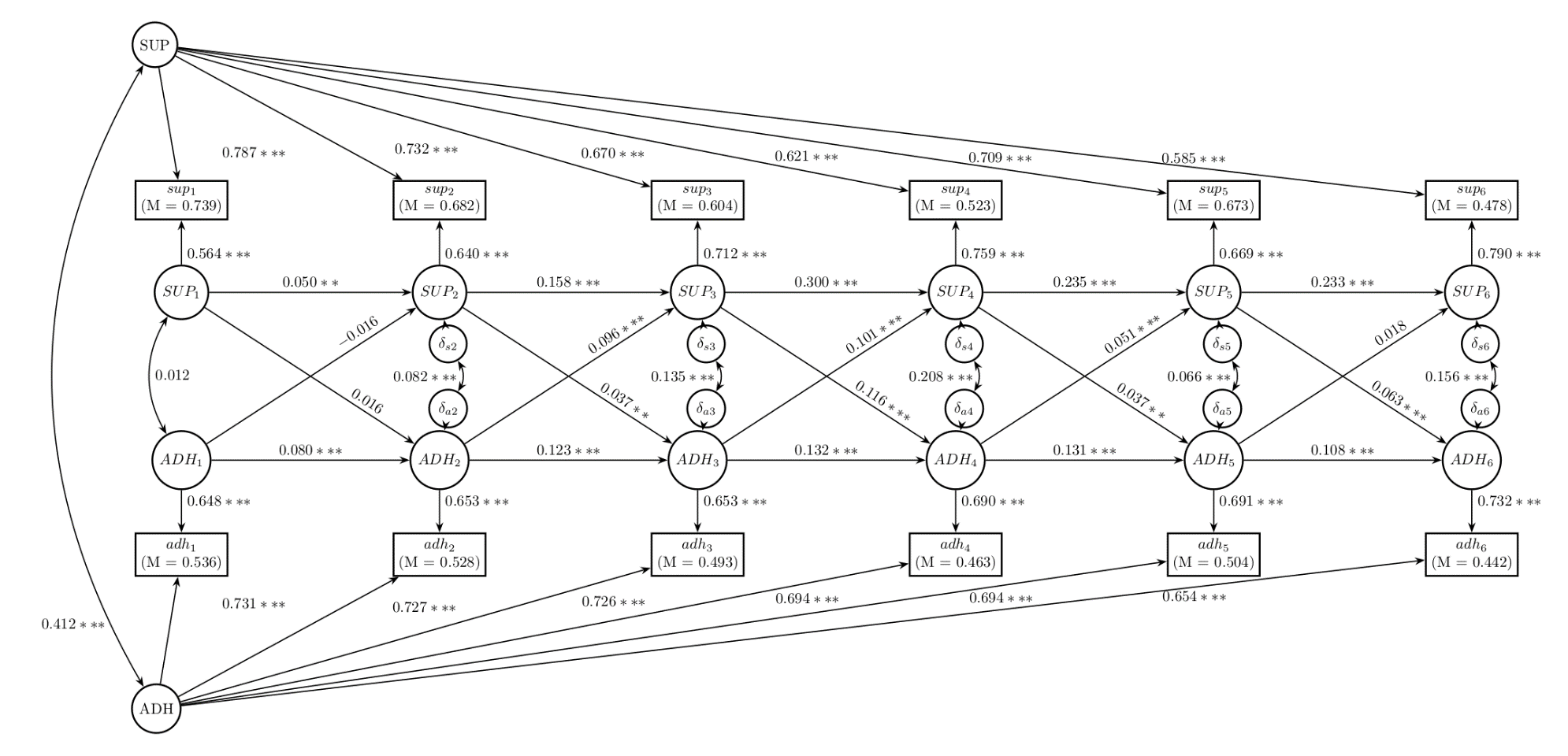


*Note:* the effects of demographic characteristics on support and adherence are not displayed.

* p < 0.05, ** P < 0.01, *** p < 0.001

*N = 20406, Chi^2^(77) = 2801.183, p = 0.000; CFI = 0.962, CFI_robust = 0.953, RMSEA = 0.042, RMSEA_robust = 0.064, SRMR = 0.046*.

# Figure S2

*RI-CLPM analysis of support and adherence regarding the policy to avoid too crowded places*


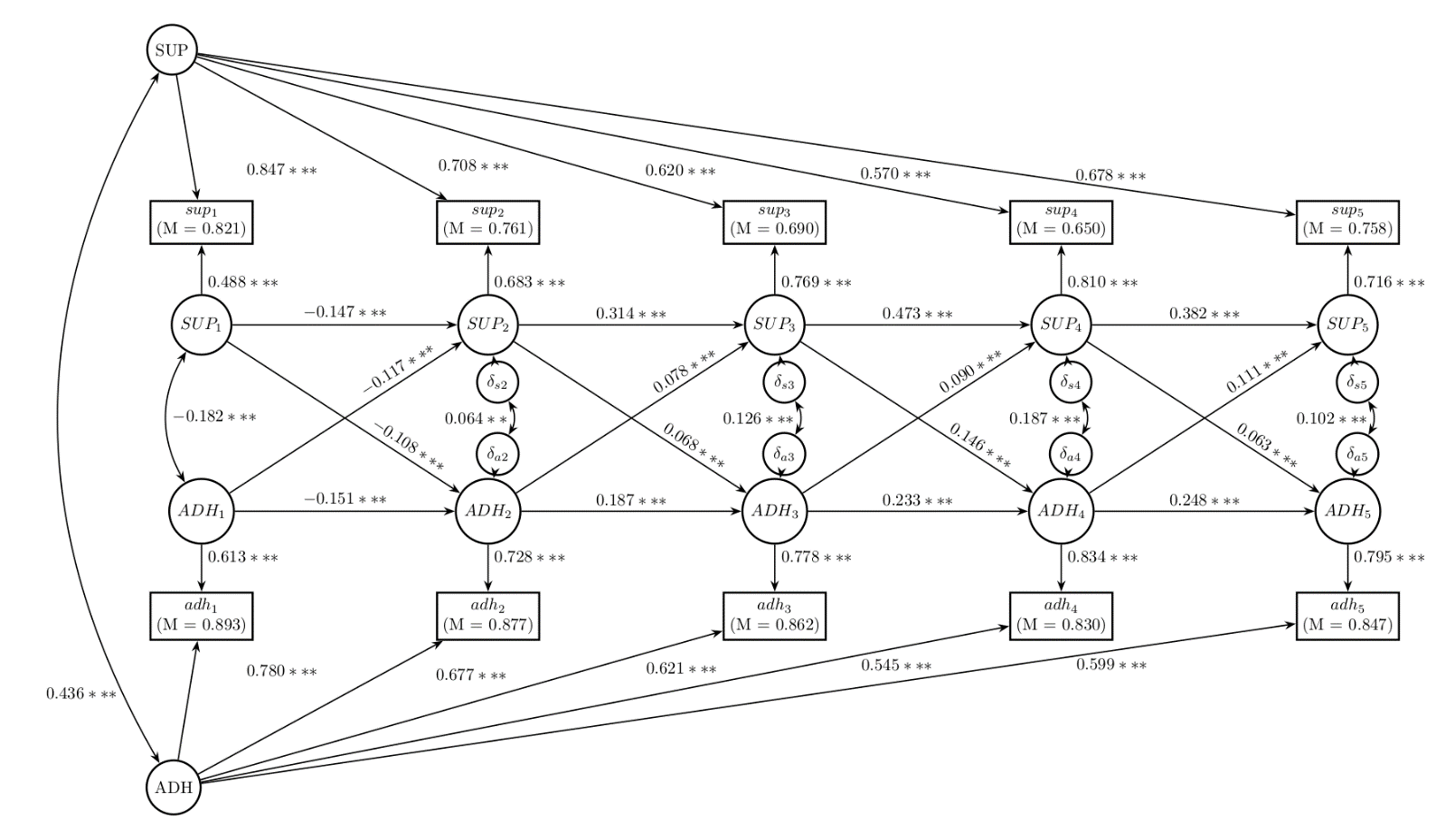


*Note:* the effects of demographic characteristics on support and adherence are not displayed.

* p < 0.05, ** P < 0.01, *** p < 0.001

*N = 19483, Chi^2^(53) = 1774.880, p = 0.000; CFI = 0.966, CFI_robust = 0.962, RMSEA = 0 .041, RMSEA_robust = 0.058, SRMR = 0.037*.

## Figure S3

*RI-CLPM analysis of support and adherence regarding wearing a facemask in public transport*


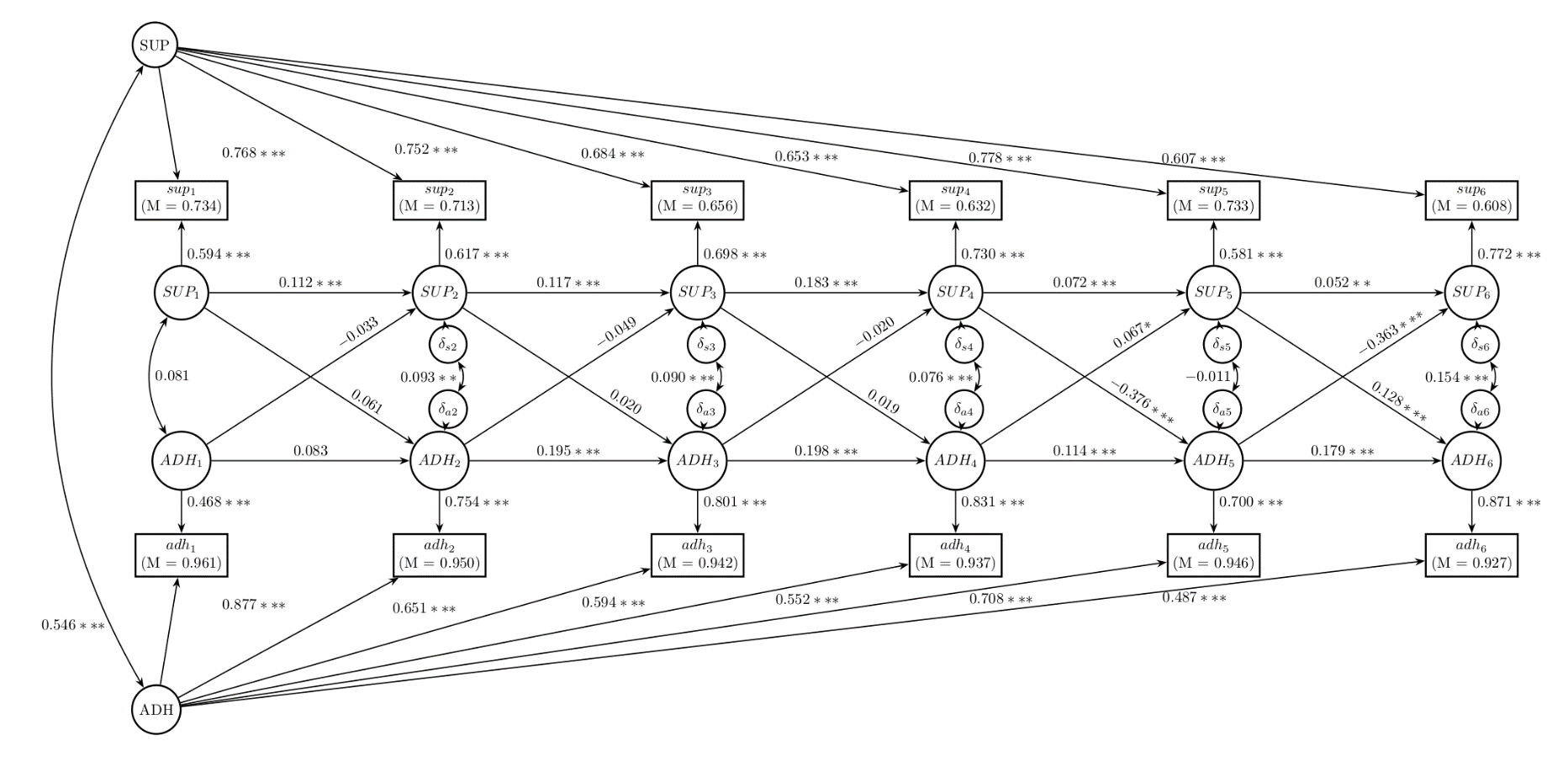


*Note:* the effects of demographic characteristics on support and adherence are not displayed.

* p < 0.05, ** P < 0.01, *** p < 0.001

*N = 20428, Chi^2^(77) = 1725.694, p = 0.000; CFI = 0.960, CFI_robust = 0.857, RMSEA = 0.032, RMSEA_robust = 0.107, SRMR = 0.091.*

## Figure S4

*RI-CLPM analysis of support and adherence regarding getting tested when having COVID-19 complaints*


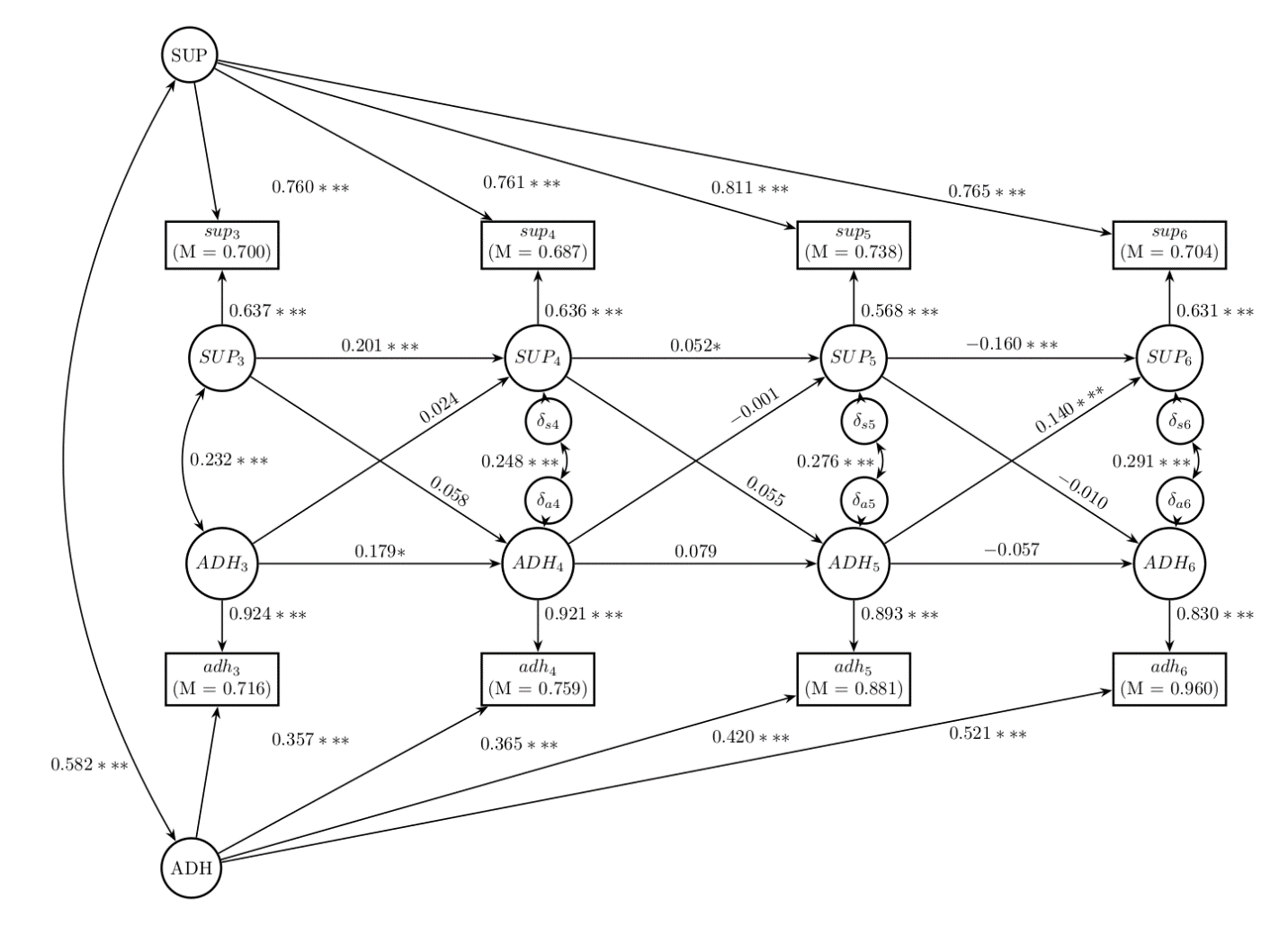


*Note:* the effects of demographic characteristics on support and adherence are not displayed.

* p < 0.05, ** P < 0.01, *** p < 0.001

*N = 15264, Chi^2^(33) = 289.888, p = 0.000; CFI = 0.988, CFI_robust = 0.984, RMSEA = 0.023, RMSEA_robust = 0.037, SRMR = 0.024.*

## Figure S5

*RI-CLPM analysis of support and adherence regarding staying at home when having COVID-19 related health complaints*


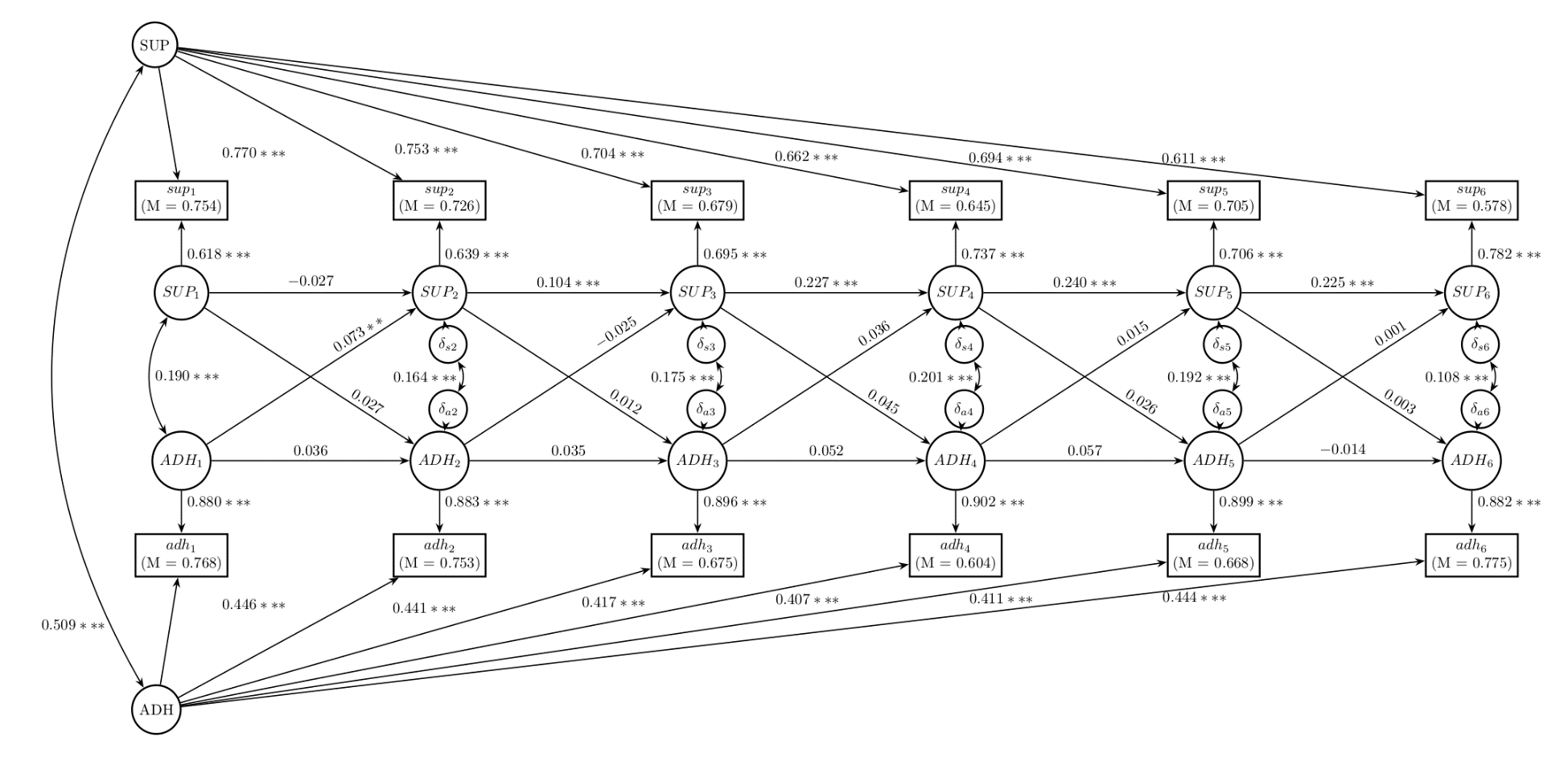


*Note:* The effects of demographic characteristics on support and adherence are not displayed.

* p < 0.05, ** P < 0.01, *** p < 0.001

*N = 20359, Chi2(77) = 1634.444, p = 0.000; CFI = 0.954, CFI_robust = 0.941, RMSEA = 0.032, RMSEA_robust = 0.055, SRMR = 0.041*

## Table S1

*Descriptive statistics of support and adherence regarding avoiding too crowded places*

|  | Support | | | | | | | Adherence | | | | | | |
| --- | --- | --- | --- | --- | --- | --- | --- | --- | --- | --- | --- | --- | --- | --- |
| Time | *M* | *SD* | *mode* | *median* | *missing* | *NA* | *N* | *M* | *SD* | *mode* | *median* | *missing* | *NA* | *N* |
| 1 | 0.92 | 0.16 | 1 | 1.00 | 0.25% | 37 | 14,625 | 0.93 | 0.14 | 1 | 1 | 0.00% | 0 | 14,625 |
| 2 | 0.85 | 0.21 | 1 | 1.00 | 0.11% | 17 | 14,880 | 0.92 | 0.17 | 1 | 1 | 0.00% | 0 | 14,880 |
| 3 | 0.79 | 0.24 | 1 | 0.75 | 0.19% | 27 | 14,193 | 0.91 | 0.18 | 1 | 1 | 3.52% | 499 | 14,193 |
| 4 | 0.75 | 0.26 | 0.75 | 0.75 | 0.24% | 29 | 11,960 | 0.87 | 0.20 | 1 | 1 | 4.90% | 586 | 11,960 |
| 5 | 0.86 | 0.21 | 1 | 1.00 | 0.17% | 22 | 12,807 | 0.89 | 0.18 | 1 | 1 | 0.00% | 0 | 12,807 |
| *Note: analytic sample* N = 19256; missingness was treated with Full Information Maximum Likelihood estimation. | | | | | | | | | | | | | | |

## Table S2

*Descriptive statistics of support and regarding keeping appropriate physical distance*

|  | Support | | | | | | | Adherence | | | | | | |
| --- | --- | --- | --- | --- | --- | --- | --- | --- | --- | --- | --- | --- | --- | --- |
| Time | *M* | *SD* | *mode* | *median* | *missing* | *NA* | *N* | *M* | *SD* | *mode* | *median* | *missing* | *NA* | *N* |
| 1 | 0.89 | 0.20 | 1 | 1.00 | 0.17% | 25 | 14,744 | 0.65 | 0.18 | 0.6 | 0.6666667 | 0.68% | 100 | 14,744 |
| 2 | 0.83 | 0.23 | 1 | 1.00 | 0.09% | 13 | 14,969 | 0.64 | 0.19 | 0.6 | 0.6666667 | 0.63% | 94 | 14,969 |
| 3 | 0.75 | 0.26 | 0.75 | 0.75 | 0.09% | 13 | 14,339 | 0.61 | 0.19 | 0.6 | 0.6000000 | 4.20% | 602 | 14,339 |
| 4 | 0.67 | 0.28 | 0.75 | 0.75 | 0.13% | 16 | 12,089 | 0.58 | 0.20 | 0.6 | 0.6000000 | 6.07% | 734 | 12,089 |
| 5 | 0.83 | 0.24 | 1 | 1.00 | 0.14% | 18 | 13,268 | 0.62 | 0.19 | 0.6 | 0.6500000 | 3.48% | 462 | 13,268 |
| 6 | 0.63 | 0.30 | 0.75 | 0.75 | 0.22% | 25 | 11,295 | 0.56 | 0.21 | 0.6 | 0.6000000 | 1.23% | 139 | 11,295 |
| *Note: a*nalytic sample N = 20453; missingness was treated with Full Information Maximum Likelihood estimation. | | | | | | | | | | | | | | |

## Table S3

*Descriptive statistics of support and adherence regarding wearing a facemask in public transport*

|  | Support | | | | | | | Adherence | | | | | | |
| --- | --- | --- | --- | --- | --- | --- | --- | --- | --- | --- | --- | --- | --- | --- |
| Time | *M* | *SD* | *mode* | *median* | *missing* | *NA* | *N* | *M* | *SD* | *mode* | *median* | *missing* | *NA* | *N* |
| 1 | 0.90 | 0.23 | 1 | 1.00 | 0.00% | 0 | 14,756 | 0.99 | 0.09 | 1 | 1 | 84.43% | 12,458 | 14,756 |
| 2 | 0.88 | 0.25 | 1 | 1.00 | 0.00% | 0 | 14,981 | 0.98 | 0.15 | 1 | 1 | 84.66% | 12,683 | 14,981 |
| 3 | 0.82 | 0.29 | 1 | 1.00 | 0.00% | 0 | 14,347 | 0.97 | 0.17 | 1 | 1 | 78.87% | 11,316 | 14,347 |
| 4 | 0.80 | 0.31 | 1 | 0.75 | 0.00% | 0 | 12,095 | 0.97 | 0.18 | 1 | 1 | 69.09% | 8,357 | 12,095 |
| 5 | 0.90 | 0.24 | 1 | 1.00 | 0.01% | 1 | 13,270 | 0.98 | 0.13 | 1 | 1 | 70.53% | 9,359 | 13,270 |
| 6 | 0.77 | 0.33 | 1 | 0.75 | 0.00% | 0 | 11,299 | 0.96 | 0.21 | 1 | 1 | 68.93% | 7,788 | 11,299 |
| *Note: analytic sample* N = 20475; missingness was treated with Full Information Maximum Likelihood estimation. | | | | | | | | | | | | | | |

Table S4

*Descriptive statistics of support and adherence regarding getting tested when experiencing COVID-19*

*related symptoms*

|  | Support | | | | | | | Adherence | | | | | | |
| --- | --- | --- | --- | --- | --- | --- | --- | --- | --- | --- | --- | --- | --- | --- |
| Time | *M* | *SD* | *mode* | *median* | *missing* | *NA* | *N* | *M* | *SD* | *mode* | *median* | *missing* | *NA* | *N* |
| 3 | 0.76 | 0.27 | 1 | 0.75 | 1.04% | 129 | 12,451 | 0.62 | 0.48 | 1 | 1 | 91.93% | 11,446 | 12,451 |
| 4 | 0.75 | 0.28 | 1 | 0.75 | 1.02% | 117 | 11,452 | 0.64 | 0.48 | 1 | 1 | 89.63% | 10,265 | 11,452 |
| 5 | 0.80 | 0.25 | 1 | 1.00 | 0.40% | 49 | 12,352 | 0.79 | 0.41 | 1 | 1 | 74.01% | 9,142 | 12,352 |
| 6 | 0.77 | 0.27 | 1 | 0.75 | 0.65% | 71 | 10,868 | 0.88 | 0.33 | 1 | 1 | 71.30% | 7,749 | 10,868 |
| *Note: analytic sample* N = 15297; missingness was treated with Full Information Maximum Likelihood estimation. | | | | | | | | | | | | | | |

Table S5

*Descriptive statistics of support and adherence regarding staying at home when experiencing COVID*

*19 related symptoms*

|  | Support | | | | | | | Adherence | | | | | | |
| --- | --- | --- | --- | --- | --- | --- | --- | --- | --- | --- | --- | --- | --- | --- |
| Time | *M* | *SD* | *mode* | *median* | *missing* | *NA* | *N* | *M* | *SD* | *mode* | *median* | *missing* | *NA* | *N* |
| 1 | 0.85 | 0.22 | 1 | 1.00 | 0.41% | 61 | 14,725 | 0.69 | 0.46 | 1 | 1 | 80.41% | 11,840 | 14,725 |
| 2 | 0.82 | 0.23 | 1 | 1.00 | 0.43% | 64 | 14,954 | 0.67 | 0.47 | 1 | 1 | 86.30% | 12,906 | 14,954 |
| 3 | 0.78 | 0.25 | 1 | 0.75 | 0.76% | 109 | 14,321 | 0.59 | 0.49 | 1 | 1 | 91.64% | 13,124 | 14,321 |
| 4 | 0.74 | 0.27 | 1 | 0.75 | 0.85% | 102 | 12,068 | 0.49 | 0.50 | 0 | 0 | 89.39% | 10,788 | 12,068 |
| 5 | 0.81 | 0.25 | 1 | 1.00 | 0.36% | 48 | 13,251 | 0.58 | 0.49 | 1 | 1 | 73.29% | 9,711 | 13,251 |
| 6 | 0.68 | 0.30 | 1 | 0.75 | 0.91% | 103 | 11,281 | 0.69 | 0.46 | 1 | 1 | 70.87% | 7,995 | 11,281 |
| *Note: analytic sample* N = 20406; missingness was treated with Full Information Maximum Likelihood estimation. | | | | | | | | | | | | | | |

**Table S6**

*Means, standard deviations, and correlations among assessments of support and adherence*

*to keeping appropriate physical distance*

|  |  | *M* | *SD* | 2 | 3 | 4 | 5 | 6 | 7 | 8 | 9 | 10 | 11 | 12 | 13 | 14 | 15 | 16 |
| --- | --- | --- | --- | --- | --- | --- | --- | --- | --- | --- | --- | --- | --- | --- | --- | --- | --- | --- |
| 1 | Support: time 1 | 0.883 | 0.199 | .638 | .577 | .507 | .585 | .452 | .272 | .293 | .261 | .273 | .219 | .239 | -.010 | .062 | -.025 | .187 |
| 2 | Support: time 2 | 0.825 | 0.230 |  | .630 | .576 | .609 | .517 | .252 | .307 | .281 | .280 | .221 | .242 | -.012 | .055 | -.004 | .187 |
| 3 | Support: time 3 | 0.747 | 0.257 |  |  | .649 | .607 | .592 | .257 | .308 | .317 | .313 | .217 | .261 | .000 | .037 | -.009 | .165 |
| 4 | Support: time 4 | 0.664 | 0.280 |  |  |  | .628 | .677 | .253 | .301 | .323 | .377 | .255 | .299 | .022 | .010 | -.013 | .217 |
| 5 | Support: time 5 | 0.814 | 0.240 |  |  |  |  | .606 | .265 | .307 | .307 | .323 | .279 | .289 | .014 | .024 | -.039 | .261 |
| 6 | Support: time 6 | 0.613 | 0.300 |  |  |  |  |  | .245 | .283 | .298 | .326 | .238 | .327 | .029 | -.008 | .003 | .263 |
| 7 | Adherence: time 1 | 0.649 | 0.179 |  |  |  |  |  |  | .604 | .573 | .528 | .528 | .471 | .049 | .006 | .024 | .221 |
| 8 | Adherence: time 2 | 0.641 | 0.188 |  |  |  |  |  |  |  | .637 | .588 | .577 | .511 | .047 | .015 | .037 | .203 |
| 9 | Adherence: time 3 | 0.606 | 0.189 |  |  |  |  |  |  |  |  | .627 | .592 | .541 | .052 | .011 | .046 | .203 |
| 10 | Adherence: time 4 | 0.576 | 0.198 |  |  |  |  |  |  |  |  |  | .601 | .560 | .055 | .002 | .040 | .207 |
| 11 | Adherence: time 5 | 0.617 | 0.196 |  |  |  |  |  |  |  |  |  |  | .553 | .054 | .009 | .028 | .196 |
| 12 | Adherence: time 6 | 0.554 | 0.207 |  |  |  |  |  |  |  |  |  |  |  | .048 | .005 | .055 | .216 |
| 13 | Education: lower | 0.110 | 0.313 |  |  |  |  |  |  |  |  |  |  |  |  | -.457 | .007 | .191 |
| 14 | Education: higher | 0.629 | 0.483 |  |  |  |  |  |  |  |  |  |  |  |  |  | .031 | -.090 |
| 15 | Gender: man | 0.361 | 0.480 |  |  |  |  |  |  |  |  |  |  |  |  |  |  | .270 |
| 16 | Age | 0.480 | 0.185 |  |  |  |  |  |  |  |  |  |  |  |  |  |  |  |

*Note:* all continuous variables were rescaled such that their values were between 0 and 1.

**Table S7**

*Means, standard deviations, and correlations among assessments of support and adherence*

*to avoiding too crowded places*

|  |  | *M* | *SD* | 2 | 3 | 4 | 5 | 6 | 7 | 8 | 9 | 10 | 11 | 12 | 13 | 14 |
| --- | --- | --- | --- | --- | --- | --- | --- | --- | --- | --- | --- | --- | --- | --- | --- | --- |
| 1 | Support: time 1 | 0.914 | 0.164 | .607 | .545 | .514 | .572 | .250 | .257 | .250 | .223 | .236 | -.024 | .062 | -.055 | .116 |
| 2 | Support: time 2 | 0.854 | 0.210 |  | .648 | .606 | .617 | .223 | .283 | .259 | .247 | .226 | -.031 | .075 | -.032 | .131 |
| 3 | Support: time 3 | 0.783 | 0.238 |  |  | .689 | .637 | .232 | .277 | .291 | .288 | .241 | -.015 | .050 | -.039 | .155 |
| 4 | Support: time 4 | 0.740 | 0.260 |  |  |  | .659 | .216 | .255 | .284 | .337 | .251 | .015 | .020 | -.025 | .231 |
| 5 | Support: time 5 | 0.848 | 0.217 |  |  |  |  | .245 | .279 | .290 | .313 | .294 | .005 | .046 | -.038 | .235 |
| 6 | Adherence: time 1 | 0.933 | 0.135 |  |  |  |  |  | .490 | .457 | .407 | .438 | -.003 | .038 | -.041 | .063 |
| 7 | Adherence: time 2 | 0.918 | 0.165 |  |  |  |  |  |  | .552 | .475 | .505 | .010 | .039 | -.040 | .093 |
| 8 | Adherence: time 3 | 0.903 | 0.177 |  |  |  |  |  |  |  | .513 | .530 | .032 | .006 | -.050 | .105 |
| 9 | Adherence: time 4 | 0.870 | 0.201 |  |  |  |  |  |  |  |  | .518 | .042 | -.020 | -.040 | .121 |
| 10 | Adherence: time 5 | 0.887 | 0.185 |  |  |  |  |  |  |  |  |  | .027 | -.002 | -.057 | .096 |
| 11 | Education: lower | 0.111 | 0.314 |  |  |  |  |  |  |  |  |  |  | -.460 | .007 | .190 |
| 12 | Education: higher | 0.629 | 0.483 |  |  |  |  |  |  |  |  |  |  |  | .028 | -.091 |
| 13 | Gender: man | 0.364 | 0.481 |  |  |  |  |  |  |  |  |  |  |  |  | .274 |
| 14 | Age | 0.483 | 0.184 |  |  |  |  |  |  |  |  |  |  |  |  |  |

*Note: a*ll continuous variables were rescaled such that their values were between 0 and 1.

**Table S8**

*Means, standard deviations, and correlations among assessments of support and adherence*

*to wearing a facemask in public transport*

|  |  | *M* | *SD* | 2 | 3 | 4 | 5 | 6 | 7 | 8 | 9 | 10 | 11 | 12 | 13 | 14 | 15 | 16 |
| --- | --- | --- | --- | --- | --- | --- | --- | --- | --- | --- | --- | --- | --- | --- | --- | --- | --- | --- |
| 1 | Support: time 1 | 0.894 | 0.238 | .655 | .580 | .556 | .610 | .511 | .269 | .274 | .312 | .299 | .341 | .312 | .030 | .002 | .007 | .210 |
| 2 | Support: time 2 | 0.874 | 0.250 |  | .621 | .595 | .631 | .517 | .249 | .294 | .301 | .326 | .336 | .339 | .012 | .020 | .013 | .175 |
| 3 | Support: time 3 | 0.815 | 0.286 |  |  | .612 | .609 | .565 | .210 | .215 | .302 | .284 | .286 | .300 | .027 | .009 | .029 | .221 |
| 4 | Support: time 4 | 0.789 | 0.307 |  |  |  | .615 | .623 | .222 | .209 | .277 | .315 | .245 | .278 | .035 | .007 | .014 | .244 |
| 5 | Support: time 5 | 0.893 | 0.242 |  |  |  |  | .580 | .169 | .231 | .284 | .321 | .341 | .352 | .024 | .018 | .007 | .244 |
| 6 | Support: time 6 | 0.761 | 0.331 |  |  |  |  |  | .134 | .172 | .217 | .269 | .237 | .314 | .054 | -.015 | .016 | .270 |
| 7 | Adherence: time 1 | 0.992 | 0.092 |  |  |  |  |  |  | .554 | .508 | .459 | .612 | .145 | -.030 | .021 | -.033 | .020 |
| 8 | Adherence: time 2 | 0.980 | 0.149 |  |  |  |  |  |  |  | .521 | .454 | .541 | .365 | -.033 | .034 | -.010 | .044 |
| 9 | Adherence: time 3 | 0.973 | 0.169 |  |  |  |  |  |  |  |  | .499 | .563 | .453 | -.016 | .025 | .008 | .080 |
| 10 | Adherence: time 4 | 0.970 | 0.182 |  |  |  |  |  |  |  |  |  | .524 | .418 | -.007 | .008 | -.009 | .071 |
| 11 | Adherence: time 5 | 0.978 | 0.143 |  |  |  |  |  |  |  |  |  |  | .549 | -.004 | .018 | .008 | .100 |
| 12 | Adherence: time 6 | 0.959 | 0.208 |  |  |  |  |  |  |  |  |  |  |  | .005 | .010 | -.007 | .129 |
| 13 | Education: lower | 0.110 | 0.313 |  |  |  |  |  |  |  |  |  |  |  |  | -.458 | .007 | .191 |
| 14 | Education: higher | 0.629 | 0.483 |  |  |  |  |  |  |  |  |  |  |  |  |  | .030 | -.090 |
| 15 | Gender: man | 0.361 | 0.480 |  |  |  |  |  |  |  |  |  |  |  |  |  |  | .269 |
| 16 | Age | 0.480 | 0.185 |  |  |  |  |  |  |  |  |  |  |  |  |  |  |  |

*Note:* all continuous variables were rescaled such that their values were between 0 and 1.

**Table S9**

*Means, standard deviations, and correlations among assessments of support and adherence*

*to getting tested when experiencing COVID-19 related symptoms*

|  |  | *M* | *SD* | 2 | 3 | 4 | 5 | 6 | 7 | 8 | 9 | 10 | 11 | 12 |
| --- | --- | --- | --- | --- | --- | --- | --- | --- | --- | --- | --- | --- | --- | --- |
| 1 | Support: time 3 | 0.761 | 0.267 | .678 | .621 | .597 | .300 | .267 | .254 | .219 | -.038 | .066 | -.091 | -.013 |
| 2 | Support: time 4 | 0.746 | 0.275 |  | .658 | .623 | .196 | .356 | .240 | .217 | -.014 | .038 | -.070 | .075 |
| 3 | Support: time 5 | 0.798 | 0.252 |  |  | .598 | .179 | .236 | .361 | .225 | -.009 | .045 | -.077 | .081 |
| 4 | Support: time 6 | 0.764 | 0.273 |  |  |  | .148 | .252 | .261 | .377 | -.021 | .049 | -.108 | .052 |
| 5 | Adherence: time 3 | 0.609 | 0.481 |  |  |  |  | .303 | .220 | .217 | -.089 | .064 | -.048 | -.160 |
| 6 | Adherence: time 4 | 0.663 | 0.474 |  |  |  |  |  | .248 | .215 | -.090 | .061 | -.169 | -.044 |
| 7 | Adherence: time 5 | 0.779 | 0.408 |  |  |  |  |  |  | .206 | -.039 | .070 | -.122 | -.083 |
| 8 | Adherence: time 6 | 0.855 | 0.327 |  |  |  |  |  |  |  | -.069 | .051 | -.159 | -.155 |
| 9 | Education: lower | 0.110 | 0.313 |  |  |  |  |  |  |  |  | -.460 | .000 | .190 |
| 10 | Education: higher | 0.631 | 0.483 |  |  |  |  |  |  |  |  |  | .035 | -.097 |
| 11 | Gender: man | 0.368 | 0.482 |  |  |  |  |  |  |  |  |  |  | .252 |
| 12 | Age | 0.495 | 0.178 |  |  |  |  |  |  |  |  |  |  |  |

*Note:* all continuous variables were rescaled such that their values were between 0 and 1.

**Table S10**

*Means, standard deviations, and correlations among assessments of support and adherence*

*to staying at home when experiencing COVID-19 related symptoms*

|  |  | *M* | *SD* | 2 | 3 | 4 | 5 | 6 | 7 | 8 | 9 | 10 | 11 | 12 | 13 | 14 | 15 | 16 |
| --- | --- | --- | --- | --- | --- | --- | --- | --- | --- | --- | --- | --- | --- | --- | --- | --- | --- | --- |
| 1 | Support: time 1 | 0.849 | 0.216 | .581 | .534 | .525 | .526 | .475 | .276 | .197 | .145 | .132 | .184 | .169 | -.006 | .033 | -.051 | .088 |
| 2 | Support: time 2 | 0.822 | 0.231 |  | .599 | .573 | .544 | .512 | .207 | .275 | .173 | .124 | .169 | .154 | -.019 | .046 | -.038 | .100 |
| 3 | Support: time 3 | 0.775 | 0.250 |  |  | .626 | .561 | .564 | .168 | .171 | .270 | .169 | .171 | .157 | -.018 | .045 | -.020 | .083 |
| 4 | Support: time 4 | 0.737 | 0.275 |  |  |  | .619 | .630 | .138 | .184 | .191 | .261 | .172 | .135 | .003 | .023 | .004 | .181 |
| 5 | Support: time 5 | 0.798 | 0.252 |  |  |  |  | .593 | .138 | .174 | .188 | .167 | .278 | .136 | .018 | .021 | -.001 | .198 |
| 6 | Support: time 6 | 0.666 | 0.302 |  |  |  |  |  | .124 | .119 | .176 | .178 | .168 | .190 | .041 | .007 | .035 | .232 |
| 7 | Adherence: time 1 | 0.686 | 0.458 |  |  |  |  |  |  | .263 | .202 | .264 | .195 | .193 | -.044 | .050 | -.186 | -.115 |
| 8 | Adherence: time 2 | 0.672 | 0.467 |  |  |  |  |  |  |  | .248 | .248 | .200 | .261 | -.099 | .079 | -.163 | -.107 |
| 9 | Adherence: time 3 | 0.610 | 0.488 |  |  |  |  |  |  |  |  | .242 | .187 | .331 | -.003 | -.002 | -.127 | -.025 |
| 10 | Adherence: time 4 | 0.526 | 0.495 |  |  |  |  |  |  |  |  |  | .222 | .064 | -.073 | .046 | -.112 | -.041 |
| 11 | Adherence: time 5 | 0.598 | 0.491 |  |  |  |  |  |  |  |  |  |  | .187 | .009 | .019 | -.075 | -.005 |
| 12 | Adherence: time 6 | 0.683 | 0.460 |  |  |  |  |  |  |  |  |  |  |  | -.058 | .062 | -.157 | -.142 |
| 13 | Education: lower | 0.109 | 0.312 |  |  |  |  |  |  |  |  |  |  |  |  | -.456 | .007 | .191 |
| 14 | Education: higher | 0.629 | 0.483 |  |  |  |  |  |  |  |  |  |  |  |  |  | .031 | -.089 |
| 15 | Gender: man | 0.361 | 0.480 |  |  |  |  |  |  |  |  |  |  |  |  |  |  | .269 |
| 16 | Age | 0.479 | 0.185 |  |  |  |  |  |  |  |  |  |  |  |  |  |  |  |

*Note:* all continuous variables were rescaled such that their values were between 0 and 1
